# Injury narrative text classification using factorization model

**DOI:** 10.1186/1472-6947-15-S1-S5

**Published:** 2015-05-20

**Authors:** Lin Chen, Kirsten Vallmuur, Richi Nayak

**Affiliations:** 1 Faculty of Science & Technology, Queensland University of Technology, 2 George St, Brisbane, Queensland, Australia; 2 Centre for Accident Research and Road Safety - Queensland, School of Psychology and Counselling, Faculty of Health, Queensland University of Technology, Victoria Park Road, Kelvin Grove, Brisbane, Queensland, Australia

**Keywords:** Narrative Text, Classification, Pre-processing, Matrix Factorization, Learning Enhancement

## Abstract

Narrative text is a useful way of identifying injury circumstances from the routine emergency department data collections. Automatically classifying narratives based on machine learning techniques is a promising technique, which can consequently reduce the tedious manual classification process. Existing works focus on using Naive Bayes which does not always offer the best performance. This paper proposes the Matrix Factorization approaches along with a learning enhancement process for this task. The results are compared with the performance of various other classification approaches. The impact on the classification results from the parameters setting during the classification of a medical text dataset is discussed. With the selection of right dimension k, Non Negative Matrix Factorization-model method achieves 10 CV accuracy of 0.93.

## Introduction

Administrative health databases, such as those collected in primary care practices, emergency departments, hospitals, mortality registration data and workers compensation databases collect various coded data and narrative text fields for the routine monitoring and analysis of injury causation and incidence. Several authors have promoted and described the value of narrative text for providing extra detail to supplement routine coded data [1,2] or for providing the required information to classify an injury post data collection if a dataset has not been coded at the point of care [3]. To develop methods for accurately classifying narrative text into injury cause categories automatically, machine learning approaches using 'gold standard' injury surveillance data can prove useful.

Methods of obtaining the information from the narrative text vary from study to study and depend on the field of research. Approaches range from basic keyword searches of text strings [4,5] through to complex statistical approaches using Bayesian methods and Natural Language Processing techniques [3,6,7]. In general, the simple approaches such as keyword search are inferior than the complicated machine learning methods in terms of achieving the specificity and sensitivity [3]. Predominantly, Bayesian model as a machine learning method is selected as the case study of using narrative text for classification of coded data [8-11,3]. Many superior classifiers such as Support Vector Machine (SVM), Matrix Factorization have never utilized in the medical-oriented dataset experiments. Considering the unique characteristics of medical narrative datasets, it is a question as to whether the conclusions drawn from the existing classification methods comparisons would still hold for a medical dataset.

Many existing works have discussed the application of matrix factorization for (1) the better visualization of dataset in 2D and 3D space, (2) clustering of documents, and (3) collaborative recommendations [12]. Only a handful of works have applied matrix factorization techniques to classification [13-15]. Predominately, the nearest neighbor approach is applied for the various tasks including classification.

Narrative text usually contains information relevant to several coded data. In this study, the aim is to map the narrative text to major injury code and external code. From the observations of dataset, it is noticed that major injury code is closely related to external code. For example, burns are associated with hot objects; lacerations are associated with sharp objects. Because of such associations existing in the dataset, the inclusion of injury code will help improve the classification accuracy of external code and vice versa.

In this paper, classification approaches on matrix factorization will be exclusively presented. The purpose of this study is as follows. (1) This study applies a series of classifiers from different classification families, including matrix factorization approaches, to a medical text narrative dataset. (2) Learning enhancement process which incorporates the injury code/external code in the feature space is studied to observe any improvement on the classification accuracy of external code/injury code. (3) Empirical comparisons of the classification family are conducted based on several evaluation metrics such as Sensitivity, Specificity, Precision, Kappa statistics and Area Under Curve (AUC/ROC), in order to evaluate the methods accurately. The best classification method is observed. (4) Results from the experiments are reported. The indications from the results and medical domain aspect are illustrated for future study.

## Literature review

Narrative text information usage includes (1) selection of cases for analysis (2) extraction of relevant information. For this work, the focus is the extraction of relevant information.

This scenario happens when the coded data is unable to identify medical cases. The basic strategy applied in some papers [4,16-18,5] to enable the selection of cases is keyword search on narrative text. Once cases were selected, the patterns and trends in injuries could then be examined by variables of interest, though no further coding of narratives was undertaken for these studies [19].

Wellman [3] focused on using injury narratives and corresponding E-codes assigned by Experts from National Health Interview Survey. A Fuzzy Bayesian model was developed for automatically assigning E-code categories to each injury narrative. It was shown that a Fuzzy Bayesian approach boosted the performance because the assumption of the algorithm is that words are conditional dependent and therefore, the combination of words increased the probability of categories.

A follow-on study of Wellman's previous Fuzzy Bayesian work combined Fuzzy and Naive Bayesian methods [10] for training and prediction of 14,000 narratives from claims from a worker's compensation insurance provider. The process of assigning code to narrative text was not fully automated when Naive Bayesian and Fuzzy Bayesian assignments disagreed, and when prediction strength is lower than a threshold.

Machine learnt algorithms for extractions of relevant information generally achieve reasonable specificity and sensitivity. Bayesian methods, even though explanatory of the results, are not necessarily the methods with the best results.

In this paper, various machine learning methods are applied to a medical dataset. Evaluations are conducted and the impacts of various factors are determined.

## Data pre-processing

Pre-processing is carried out to ensure the quality of data. Those records with a null entry in injury description are removed during the Data Removal step. Inconsistencies in External Cause Code and Major Injury Factor Code due to different coding systems being adopted at different periods of time are also corrected.

**Misspelling: **Misspelling can happen frequently in busy medical settings [20]. Failure to detect misspelling results in poor classification. Due to its severe consequences, research in spelling auto-correction is well-studied. The early researchers utilise the single edit operation such as insertion, deletion, and substitution, for the words that do not appear in a dictionary [21]. More recently, statistical models such as error models and n-gram language models are employed, especially in online query spelling correction settings [22]. In the medical environment, most misspellings are caused by mistyped keys and misunderstanding of the correct spelling of words [20]. We have corrected the misspellings by finding and replacing the misspelt word with a soundalike word, which is the smallest edit distance from the original word. We utilised Aspell for the soundalike type of spelling correction as the performance of Aspell has been reported better than Ispell or Google (Atkinson, 2006).

**Removal of punctuation: **Punctuation contains no actual meaning of information and can consume extra storage space in the lengthy document-term entry, if they are not removed. The removal of punctuations should be performed before stop-word removal and stemming procedures, otherwise, they may not be performed accurately. The words before and after the punctuation are identified as a single word. For example, 'fall (from the high chair)'. If punctuation is not removed before the stop word removal, 'from' is identified as a word, thus, the stop-word 'from' is not removed.

**Phrases and uniformity of abbreviation: **The text in the field 'External Cause' often contains phrases. If the words in a phrase are processed independently, the phrase may lose the actual meaning. For example, 'fall off' will become 'fall' after the stop-word removal of 'off'. Similarly, many abbreviations have many forms of entry. For example, 'kph' and 'kmph' both are associated with kilometre per hour. All the abbreviations with many variation forms in the text narratives are identified and only one form of abbreviations is used.

**Feature reduction: **Stop-word removal and Stemming are common steps to reduce the number of terms in the document-term entry before text mining is carried out. In this work, the Porter stemming algorithm is utilized. Additionally, terms that appear too infrequently or too frequently should be removed. Infrequent terms only appear in a few text narratives and are not applicable to the majority of records. Frequently appeared terms should be removed as they are like stop words. If the frequent terms appear in all the classes, they are not good text indicators for classifications and thus serve no purpose of keeping the term. The results for frequent terms are discussed later in the section entitled 'Impact of removal of topn terms'. We found that frequent term should be removed with the guidance of medical experts as the removal of Top-N terms without guidance leads to the worse accuracy. Medical experts know which terms are the unique to some classes and which terms are quite general and appear in lots of classes.

**Figure 1:**
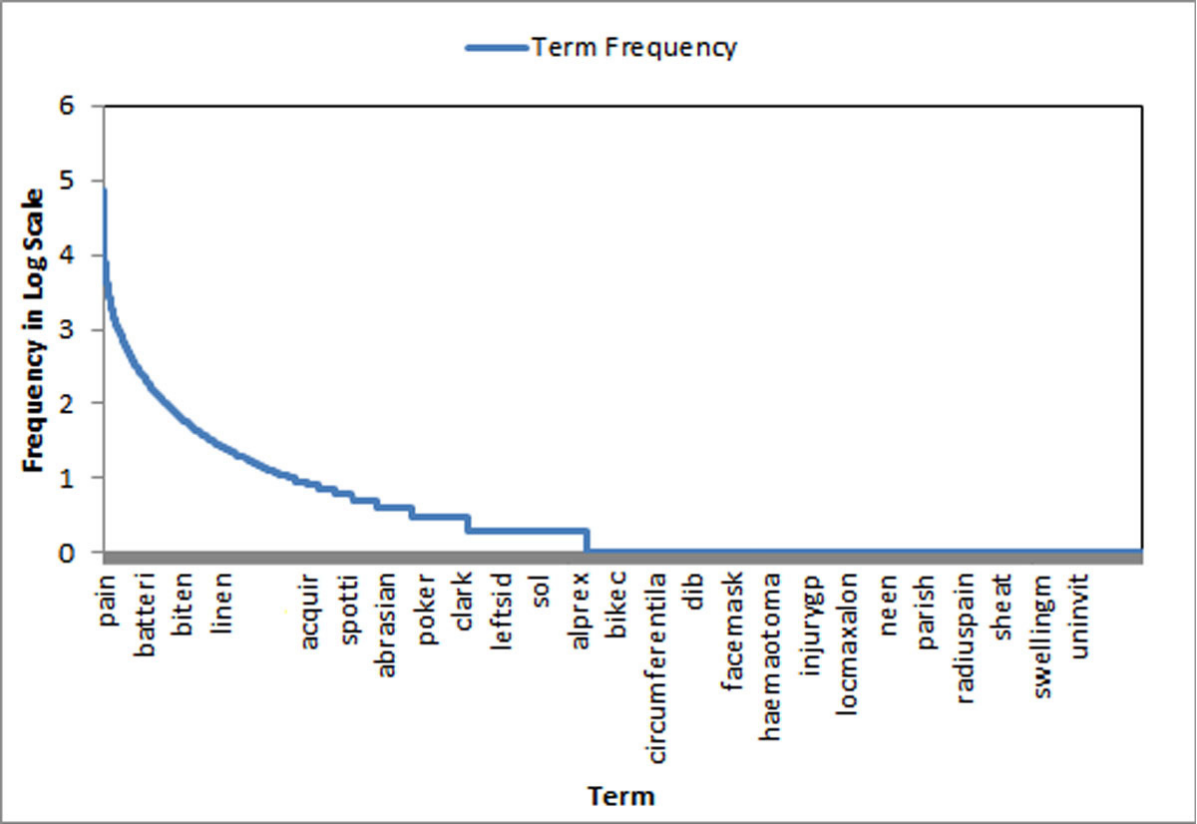
**Term frequency in log scale**.

Figure 1 shows that a few terms appear frequently, a reasonable number of terms appear moderately and many terms appear rarely. In order to decide the cut-off criteria for differentiating infrequently and frequently appearing terms, an EM clustering approach is employed [23].

Let *x_i _*represent the frequency of term *i *in the dataset. Let *X = (x*_1_*, x*_2_*,..., x_n_) *represent all the terms frequency in the dataset. Let the dataset be divided into two subsets-frequent term subset and infrequent term subset. Given the dataset X, the objective is to mine a set of parameters *θ *= {*θ*_1_*, θ*_2_} such that the probability *P *(*X|θ*} is maximized where *θ_j _*= (*u_j, _σ_j_*) are the mean and standard deviation. By iteratively applying E-step and M-step, the parameters are calculated and therefore the clusters can be decided.

In E-step, the probability of *x_i _*belonging to each distribution can be calculated by using

(1) $$P\left(\theta_{j}|x_{i},\theta \right)=\frac{P\left(x_{i}|\theta_{j}\right)}{\sum_{m=1}^{2}P\left(x_{i}|\theta_{m}\right)}$$

In M-step, the parameters are adjusted by using

(2) $$u_{j}=\frac{1}{2}\frac{\sum_{i=1}^{n}x_{i}P\left(\theta_{j}|x_{i},\theta \right)}{\sum_{i=1}^{n}P\left(\theta_{j}|x_{i},\theta \right)}$$

(3) $$\sigma_{j}=\sqrt{\frac{\sum_{i=1}^{n}P\left(\theta_{j}|x_{i},\theta \right){\left(x_{i}-u_{j}\right)}^{2}}{\sum_{i=1}^{n}P\left(\theta_{j}|x_{i},\theta \right)}}$$

After each iteration, the convergence test is performed. When *θ*^N ^*− θ*^*N −*1 ^<*∈ *is satisfied, the iteration process stops. *∈ *is set to 1.0e-6.

The total number of terms was 27,734 initially and this is cut down to 4,924 after feature reduction.

## Matrix factorization based classifier

The medical narrative text injury dataset is composed of many short documents. Each document describes the injury events such as how it happened, where it happened and what caused it. The data can be characterized as high-dimensional sparse, in which few features are irrelevant, but features tend to be correlated with one another and generally organized into linearly separable categories. Because of this characteristic, it is more appropriate to use matrix factorization techniques mapping all the features to lower dimensional space rather than reduce the number of features. Feature reduction methods such as Mutual Information, Chi-Squared criteria and odds ratio aim to reduce the number of features. However, no single feature selection method can work well with all text categorization methods, as different classifiers evaluate the important features differently. Simply removal of features may cause of the removal of important features for some classifiers. On the other hand, feature extraction such as matrix factorization should work well as all the features are kept and transformed into lower dimensional space. By doing so, noise is reduced and the latent semantic structure of the vocabulary used in the dataset becomes more obvious, thus, the lower-dimension feature space improves the classification performance. We propose to use two matrix factorization techniques, Singular Value Decomposition (SVD) and Non Negative Matrix Factorization (NNMF).

### Singular value decomposition (SVD)

Let *D *be identified as the narrative text injury dataset. Singular Value Decomposition transforms an original matrix *D *∈ *R^m×n ^*into left singular matrix *U *∈ *R^m×m^*, right singular matrix *V *∈ *R^n×n ^*and diagonal matrix ∑ ∈ *R^m×n^*. Formally, *D *= *U *Σ*V^T^*. Let the approximation denoted as ${\tilde{D}}_{k}$ which is formed by considering only the top *k *singular value in Σhas the smallest distance to *D *measured in any unitarily invariant norm [12]. More specifically, ${\tilde{D}}_{k}$ can be decomposed to ${\tilde{D}}_{k}=U_{k}\Sigma_{k}V_{k}^{T}$.

Where *k *is the *k *largest singular values, *U_k _*∈ *R^m×k^, V_k _*∈ *R^n×k^*, and ∑*_k _*∈ *R^k×k^*.

**Model-based Classification: **Let *D *be identified as the narrative text injury dataset containing *m *number of documents. Let SVD approximation of narrative text documents identified as ${\tilde{D}}_{k}$, ${\tilde{D}}_{k}=\left({\tilde{d}}_{1},{\tilde{d}}_{2},\ldots ,{\tilde{d}}_{m}\right)$. For each document ${\tilde{d}}_{j}$, it can be represented by *k *largest singular values as ${\tilde{d}}_{j}=\left(w_{1_{j}},w_{2_{j}},\ldots ,w_{k_{j}}\right)$.

The classification problem is to approximate a target function $f:{\tilde{D}}_{k}\to C$ where *C *= (*c*_1_, *c*_2_...*c_x_*) is a set of pre-defined categories. In this paper, various families of classifiers are tested in the experiment section, with only the best classifier being applied on the approximated document matrix ${\tilde{D}}_{k}$.

**Memory-based Classification**: Inspired by the CMF method [13], the approximation matrix can be represented as

(4) $${\tilde{D}}_{k}=U_{k}\Sigma_{k}V_{k}^{T}=U_{k}\Sigma_{k}V_{k}^{T}V_{k}V_{k}^{T}=DV_{k}{V^{T}}_{k}$$

Because *V_k _*is singular matrix, $V_{k}^{T}V_{k}=I$ where I is identity matrix.

Let the training dataset be denoted as ${\tilde{D}}_{k}^{Train}$ and let the testing dataset be denoted as ${\tilde{D}}_{k}^{Test}$. The singular vectors $V_{k}^{Train}$ extracted from training phase are also the representative of the test data as training data and test data should exhibit the similar characteristics [13].

(5) $${\tilde{D}}_{k}^{Train}=D^{Train}V_{k}^{Train}{\left(V_{k}^{Train}\right)}^{T}$$

(6) $${\tilde{D}}_{k}^{Test}=D^{Test}V_{k}^{Train}{\left(V_{k}^{Train}\right)}^{T}$$

By comparing the similarity of each document in ${\tilde{D}}_{k}^{Test}$ and the document in ${\tilde{D}}_{k}^{Train}$, the most likely class is assigned to the document in ${\tilde{D}}_{k}^{Test}$ with the class label from the most similar document in ${\tilde{D}}_{k}^{Train}$. Formally described as,

(7) $$C=\text{arg max}\left\{\frac{{\left({\tilde{D}}_{k}^{Train}\right)}^{T}{\tilde{D}}_{k}^{Test}}{║{\tilde{D}}_{k}^{Train}║║{\tilde{D}}_{k}^{Test}║}\right\}$$

### Non-negative matrix factorization (NNMF)

It is more natural to use NNMF rather than SVD as each document is the addition of topics and therefore the coefficients should be all non-negative values. Further, topics under a document are not completely independent of each other and therefore, the semantic space capturing the topics are not necessarily orthogonal [24].

Given *D *∈ *R^m×n^*, it can be decomposed to *A *∈ *R^m×r ^*and *H *∈ *R^r×n ^*where *r *is the new feature space [25]. Formally,

(8) D≈AH

The approximation of equation can be solved by either applying Kullback-Leiber(KL) divergence or Forbeius norm [25]. *A *is interpreted as a representation of documents in the newly formed space and H is representation of terms in newly formed space.

**Model-based classification: **Because *A *is deemed to be the representation of documents in the newly formed feature space, A is used in classification. Let *A^Train ^*be identified as the document representation from *D^Train^*. Let *A^Test ^*be identified as the document representation from *D^Test^*. During the training phase, the best classifier is selected for training *(A^Train^, C^Train^) *which is the class label for each training case. During the testing phase *A^Test ^*is supplied to the trained classifier for the prediction of the class label for each testing case.

**Memory-based classification**: Let the training dataset be denoted as ${\tilde{D}}_{k}^{Train}$ and let the testing dataset be denoted as ${\tilde{D}}_{k}^{Train}$

(9) $${\tilde{D}}_{k}^{Train}\approx A^{Train}H^{Train}$$

(10) $${\tilde{D}}_{k}^{Test}\approx A^{Test}H^{Test}$$

By comparing the similarity of each document in *A^Train ^*and the document in *A^Test^*, the most likely class is assigned to the document in *A^Test ^*with the class label from the most similar document in *A^Train ^*More specifically,

(11) $$C=\text{arg max}\left\{\frac{{\left(A^{Train}\right)}^{T}A^{Test}}{∥A^{Train}∥∥A^{Test}∥}\right\}$$

## Learning enhancement

From the observations of dataset, it is noticed that major injury code is closely related to external code. For example, burns are associated with hot objects; lacerations are associated with sharp objects. Suppose the classification assignment of major injury code/external code can be represented as supplementary features for the classification of external code/major injury code.

Let the k features denoted as *F = (f*_1_, *f*_2_...,*f*_*k*_) with each feature representing as a text. Suppose there is *p *number of class of external code. Let the external code class represented as supplementary features and the features are changed to *F = (f*_1_, *f*_2_,... *f*_*k*, _*f*_*k*+1_, *f*_*k*+2_,... *f*_*k*+*p*_). If the instance belongs to class *i*, then only *f*_*k*+*i *_has value 1 and the rest of class represented as features *f*_*k*+1_, *f*_*k*+2_....*f*_*k*+*p *_has value as 0. The objective is to classify the major injury code using the newly changed features F. The classification of external code can be done similarly using the major injury code as the supplementary feature.

## Experimental setup

Our dataset contains 15,000 emergency department presentation records from a sample of hospitals collecting data for the Queensland Injury Surveillance Unit during 2009 to 2012. Each record contains narrative text field which is the description of various aspects of medical case including the cause and mechanism leading to the injury, activity involved, absence or presence of protective equipment and treatment of the case. The External Cause is the primary event, circumstances or condition associated with the occurrence injury. Major Injury Factors are the types of objects and substances involved in occurrence of injury. They have been coded by professional medical staff using ICD-10 classification system.

**Figure 2:**
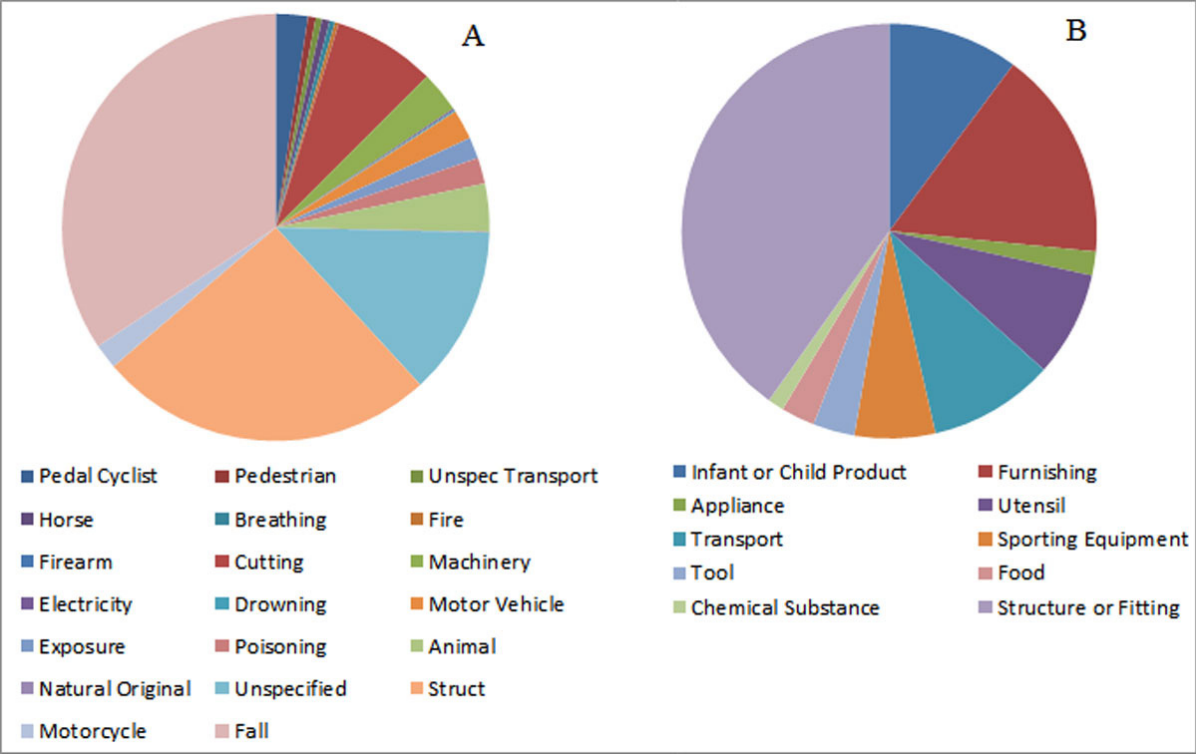
**Distribution of dataset**. A) shows the external code distribution and B) shows the injury factor code distribution.

**Dataset**: The objective of experiments is to evaluate the performance of various classifiers on the injury narrative dataset. Table 1 shows the statistics of the injury narrative dataset. Documents with empty injury description text, or empty External Cause class, or empty Major Injury Factor Cause Class are not included in the dataset. Figure 2 shows that the dataset is imbalanced, with two major classes taking up about 70% of the whole population for External Code distribution, while two major classes in Injury Factor take up about 50% of the population.

**Table 1:** Dataset statistics.

Dataset	# Document	Average Length	Max Length	Min Length
Training Dataset	10,000	70	254	1

Testing Dataset	5,000	68	245	1

**Evaluation criteria**: The criteria used are designed for the medical domain. To avoid bias because of the imbalanced dataset, Kappa statistic is employed. Note TP in Table 2 corresponds to true positive which is the items correctly classified and belongs to the positive class. FP is false positive which refers to the items incorrectly classified and should belong to negative class. TN is true negative which refers to item correctly classified and belongs to the negative class. FN is false negative which means the item is misclassified and belongs to positive class.

**Table 2:** Evaluation measures.

Measure	Definition	Equation
**Positive Predictive Value (PPV)**	Ratio of truth positive to number of cases truly falls under the classes	TP/(TP+FP)

**Sensitivity**	Proportion of actual positives which are correctly identified	TP/(TP+FN)

**Specificity**	Proportion of negative which are correctly identified	TN/(FP+TN)

**10 Cross Validation Accuracy**	Split the dataset into 10 folds and runs classification 10 times	(TP+TN)/(TP+FP+TN+FN)

**Kappa**	Robust measure taking into account the agreement by chance	K = (I*_o_*-I*_e_*)/(1-I*_e_*) I*_o _*= (TP+TN)/(TP+TN+FP+FN) I*_e _*= ((TN+FN)(TN+FP)+ (TP+FP)(TP+FN))/n^2^ n = TP+TN+FP+FN

**Area Under Receiver Operating Characteristic Curve (AUC)**	Identify the relation between sensitivity and specificity	Area under curve plotting sensitivity and (1-specificity)

### Traditional classifiers

**Decision tree: **C4.5 algorithm is employed and a pruning approach is adopted for reducing the complexity of the classifier and improving the accuracy.

**Probabilistic: **Naive Bayes algorithm with multi-variate Bernoulli model is used. This is known for its performance when the dataset contains small vocabularies [26].

**Linear: **Support Vector Machine (SVM) is used with the radial basis function selected as the kernel type and regression is employed as SVM type.

**Neural network: **A two-layer neural network is trained with the expected output and actual output in order to get the weights for each term feature. A backpropogation algorithm is utilized in order to minimize the error.

**K-NN: **The nearest neighbour approach in which k = 1 is adopted.

**Boosting: **Adaboost with Decision Tree as the weak learner is applied in our experiments. Boosting-based methods are considered superior to Ensemble-based (Majority Voting) methods which can suffer from the overfitting problem [27].

## Results

### Impact of classifier

**Figure 3:**
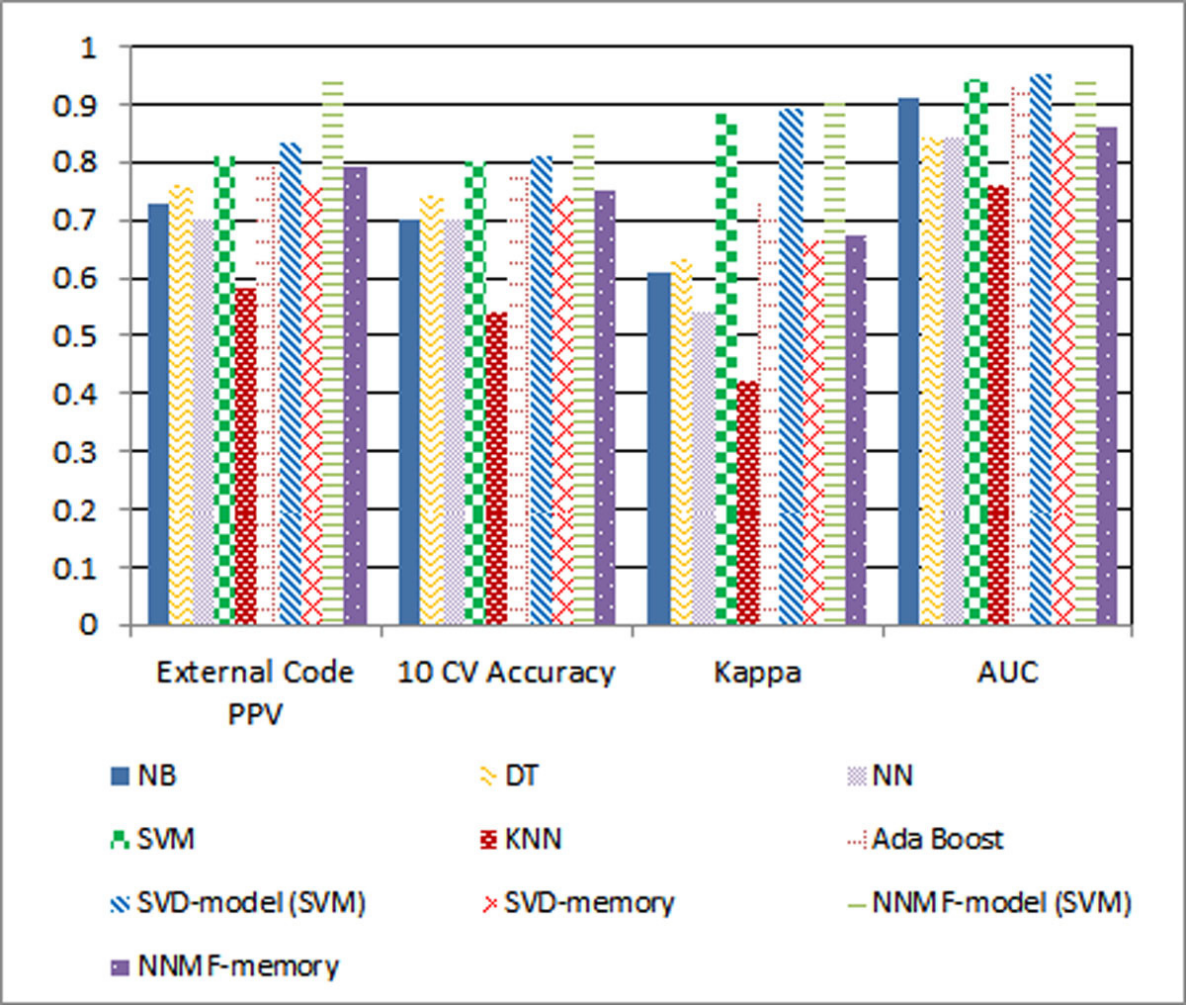
**External cause classification**.

**Figure 4:**
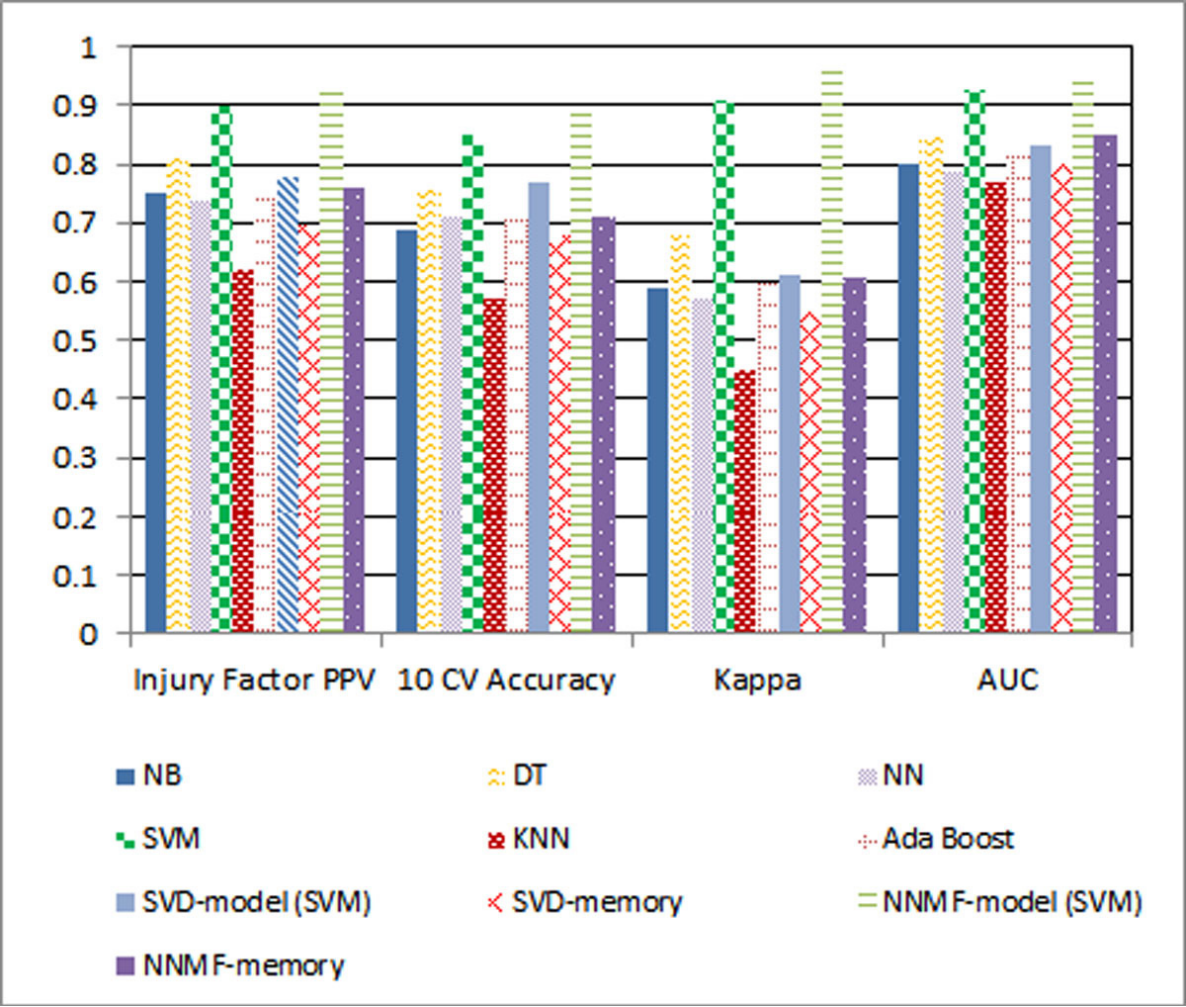
**Injury factor classification**.

**General performance: **The NNMF-model (NNMF with SVM) achieved the best performances against all the evaluation criteria for both External Code and Injury Factor Code classifications. The experiments show that NNMF can achieve better performance than SVD for the text classification because NNMF captures the characteristics of text where the topics should be related in additive manner rather than being independent in an orthogonal manner. Without the boost of Matrix Factorization, SVM achieves lower results. But SVM can achieve better results than all of the tested classic methods because SVM is highly tolerant to sparse and high dimensional medical injury text dataset with correlated features. Comparing KNN to the rest of the classic approaches, SVD-model to SVD-memory, NNMF-model (NNMF with SVM) to NNMF-memory, model-based approaches are superior to memory-based approaches. This is because the memory-based approach treats each feature equally and cannot differentiate the importance of features for each class. Figures 3 and 4 summarise the classification performance of the various tested classifiers.

**Kappa statistic: **Kappa statistic measures the agreement between automated classification results and the gold-standard class assignment by medical expert(s). A value with ranges from 0.21-0.4 and 0.41-0.6 are associated with fair and moderate agreement respectively, while between 0.61 and 0.8 show substantial agreements [28]. Even though the dataset shows biased distribution, most classification methods except KNN still can achieve substantial agreement with the gold-standard. This means the agreement that would occur by chance is generally low.

**AUC: **Despite the imbalanced dataset, AUC results are consistent with 10 CV Accuracy. According to AUC results, NNMF-model (NNMF-SVM) achieves the best performance for both External Cause and Major Injury Factor classification.

Prediction of external code and major injury factor

For most classes, sensitivity, specificity, and PPV of External cause and Major Injury Factor achieved high results as Table 3 and 4 indicated. Some classes (i.e. Fire, Firearm, Cutting, Machinery, Chemical Substance) achieved poor results. The reason behind is the lack of sufficient training cases associated with these classes. Increasing the number of training cases for these classes may help improve the performances.

**Table 3:** Performances of major injury code.

Class Description	Sensitivity	Specificity	PPV
Infant	0.89	0.91	0.87

Furnishing	0.90	0.83	0.90

Appliance	0.86	0.98	0.81

Utensil container	0.90	0.91	0.93

Transport	0.94	0.90	0.95

Sporting equipment	0.92	0.95	0.92

Tool	0.92	0.90	0.86

Food	0.86	0.97	0.74

Chemical substance	0.70	0.98	0.65

Structure fitting	0.90	0.93	0.92

**Table 4:** Performances of external code.

Class Description	Sensitivity	Specificity	PPV
Motor vehicle	0.94	0.92	0.92

Motorcycle	0.90	0.93	0.90

Pedal cyclist	0.90	0.95	0.93

Pedestrian	0.77	0.98	0.57

Unspecified transport	0.73	0.98	0.21

Horse related	0.94	0.92	0.95

Animal related (exclude horse)	0.92	0.93	0.89

Fall	0.84	0.95	0.93

Drowning submersion	0.90	0.92	0.64

Breathing	0.48	0.97	0.36

Fire	0.64	0.98	0.66

Exposure	0.85	0.91	0.89

Poisoning	0.80	0.93	0.82

Firearm	0.6	0.98	0.18

Cutting	0.50	0.98	0.81

Machinary	0.63	0.98	0.73

Electricity	0.92	0.92	0.89

Natural original	0.85	0.96	0.66

Unspecified	0.80	0.95	0.51

Struck	0.81	0.95	0.82

### Impact of training size

**Figure 5:**
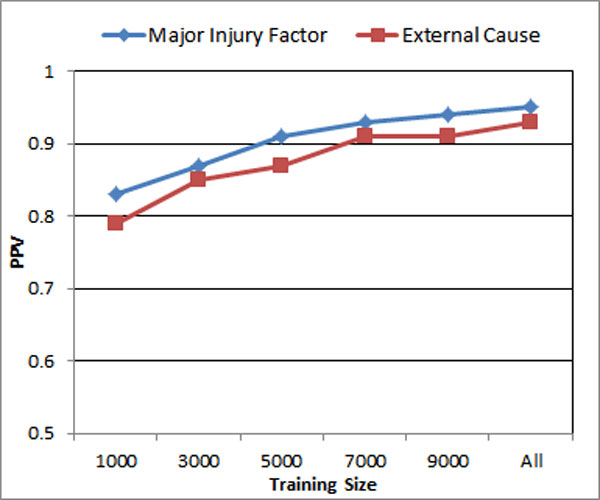
**Impact of training size**.

**Figure 6:**
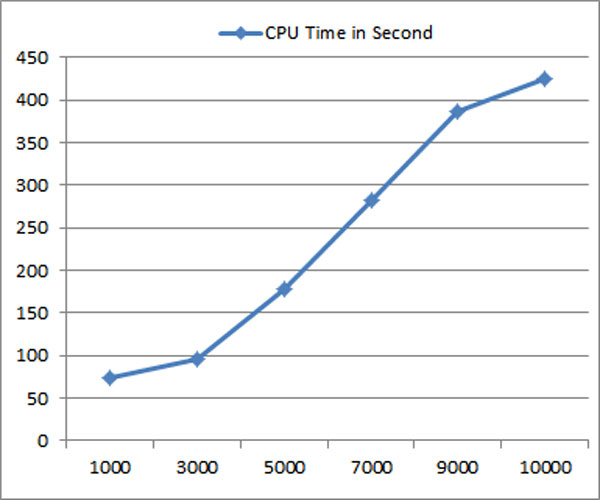
**Impact of training size against computation time**.

Figure 5 shows the impact of training size in terms of predicting Major Injury Factor and External Cause in the testing dataset. The two lines show a similar trend on the graph. With the increase in training size, PPV result improves. However, the improvements of PPV are clearly slowing down as the increase of the training size continues. Increasing the training size usually leads to higher computational cost. Figure 6 shows that increase of training size leads to much higher increase in computation time. The balance of increasing the training size for the purpose of better performance and the increase of computational resources should be decided with the consideration of the ratio of the increase of PPV/accuracy to the increase of computation costs.

### Impact of removal of TopN terms

**Figure 7:**
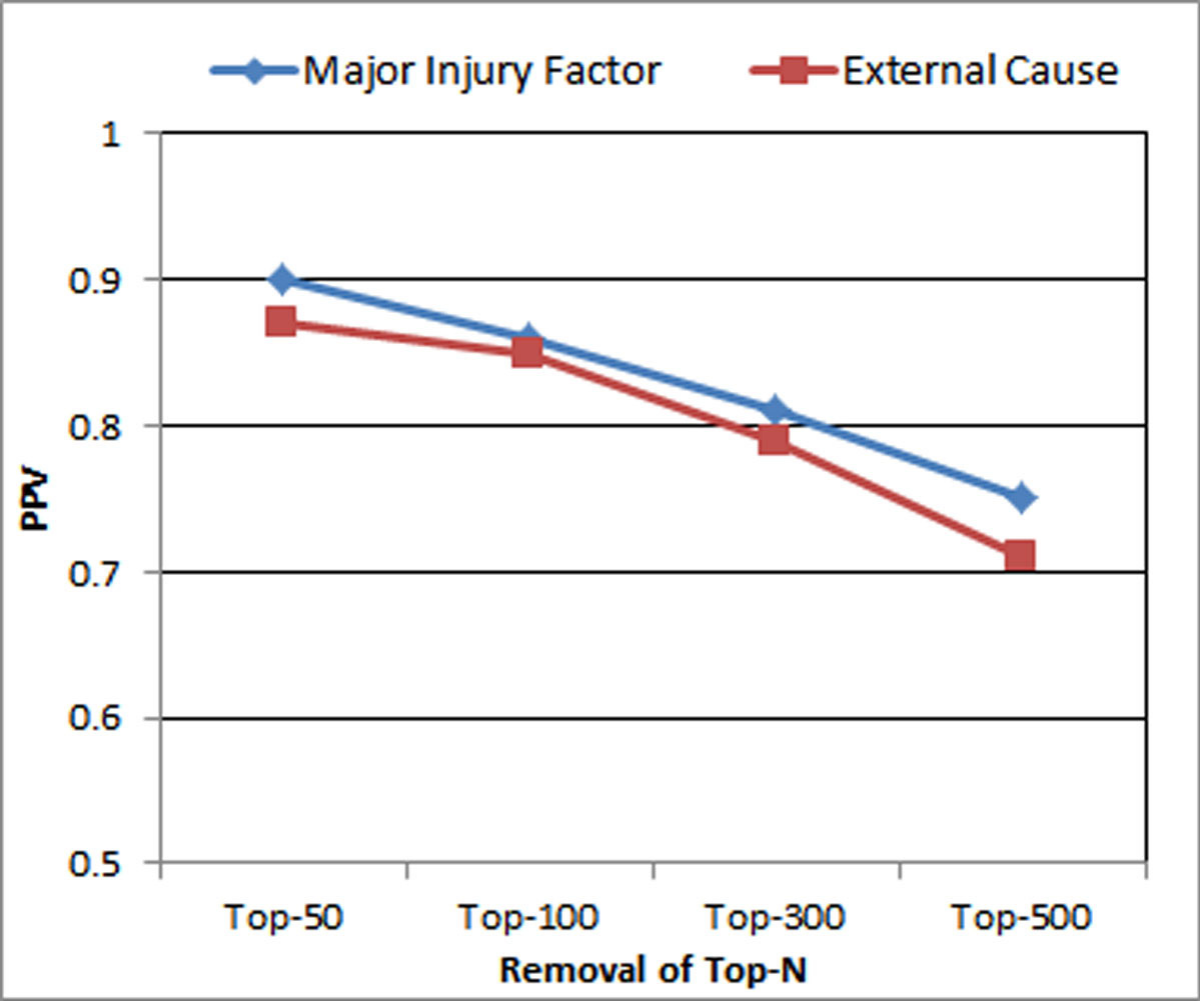
**Impact of removal top-n**.

TopN terms usually are associated with frequently appearing terms. These common terms appear regularly in all the classes and they are not deemed as distinguishing identifiers. However, the empirical results shown in Figure 7 indicate removal of TopN terms decreases the precision rate on the testing dataset. It is possible that some frequently appearing terms do associate with class identifiers and their removal can cause the performance to decrease. Guidance from medical experts for the removal of TopN terms is desirable in future.

### Impact of term weights

**Figure 8:**
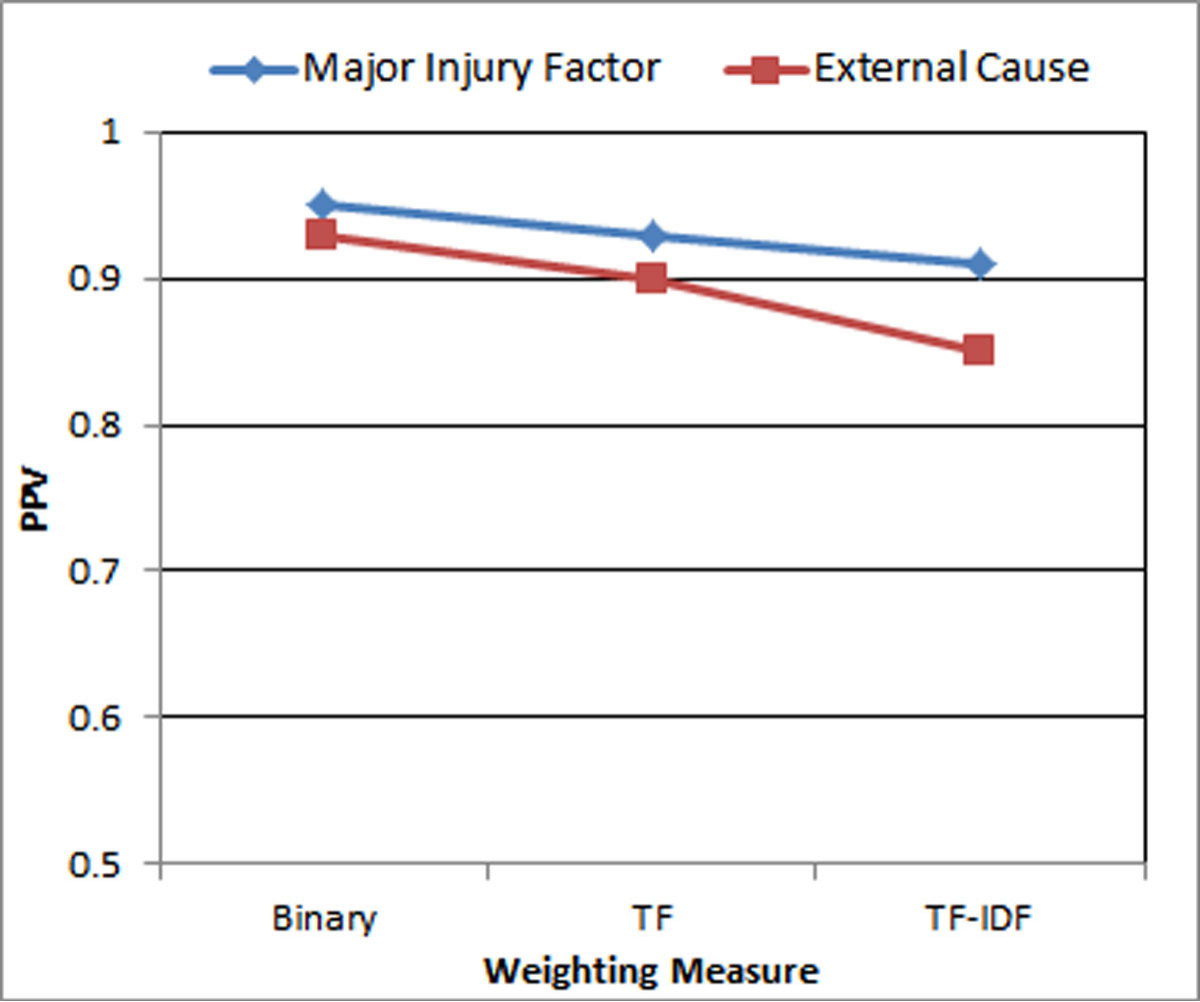
**Impact of weighting measure**.

Figure 8 illustrates that the best PPV is achieved when Binary weighting is applied followed by TF and then TF-IDF. BM25 is not tested as it works the best with lengthy document and our dataset contains lots of short documents. Surprisingly, TF-IDF does not achieve the best result. This might be due to the length of the documents in the dataset. When the document is long, TF-IDF usually works well as TF part identifying the important terms within a document and the IDF part identifying the unique terms which is different from other documents in the dataset. In injury text narrative dataset, the length of each document is short which explains the lower performance accounting from the term frequency for each document. In this experiment, how much influence the IDF part played is not known. In future, it might be worth trying to use the weighting score which accounts both the appearance of the term (term appears or not) and IDF.

### Impact of k selection in factorization

**Figure 9:**
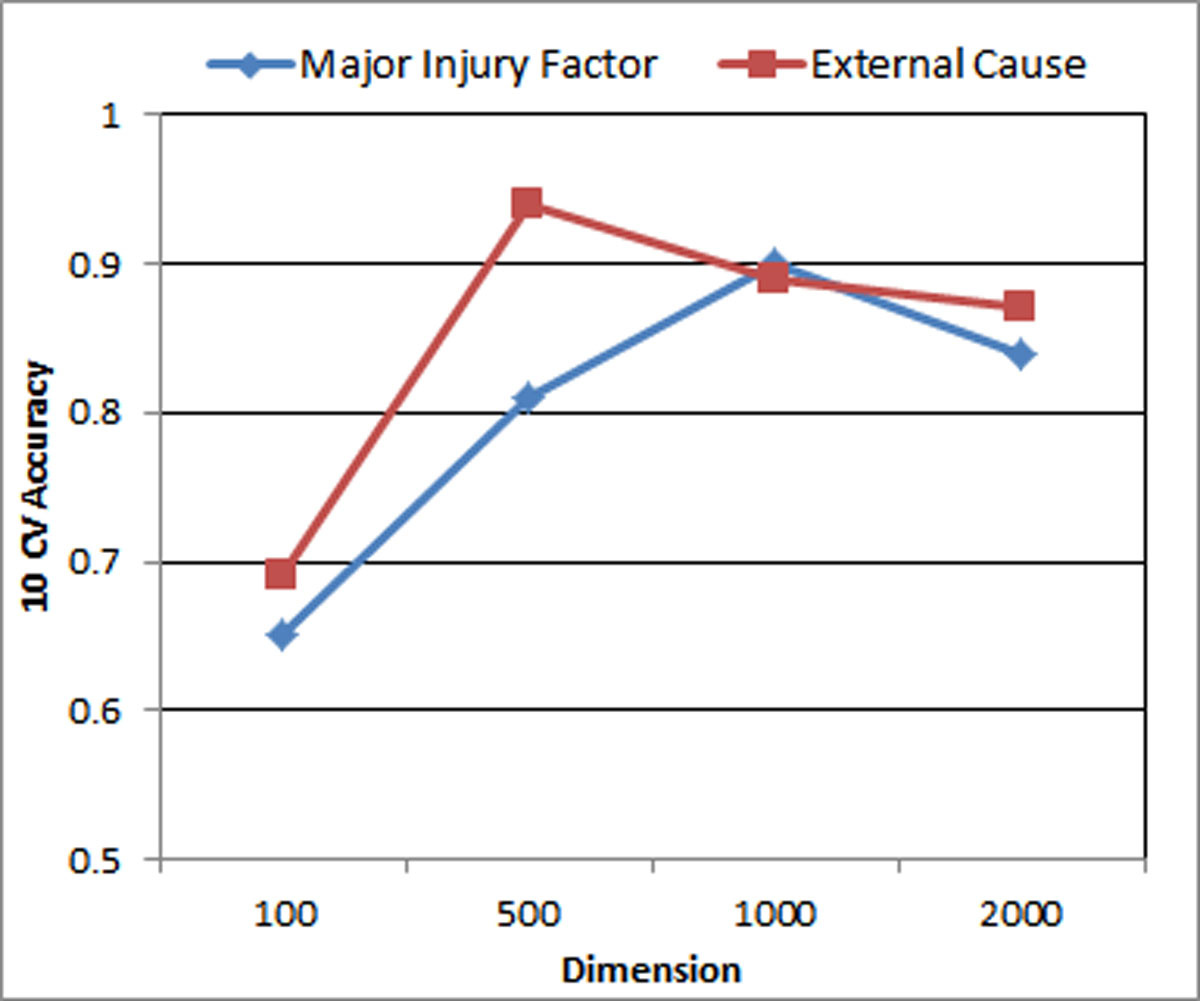
**Impact of k selection**.

With the increase of k, the accuracy scores for both classes increase initially and then the performance drops after some threshold. The performance reaches its climax at *k = 500 *for External Cause Code, while Major Injury Factor achieves a climax at *k = 1000*. This is shown in Figure 9.

### Impact of enhancement learning

**Figure 10:**
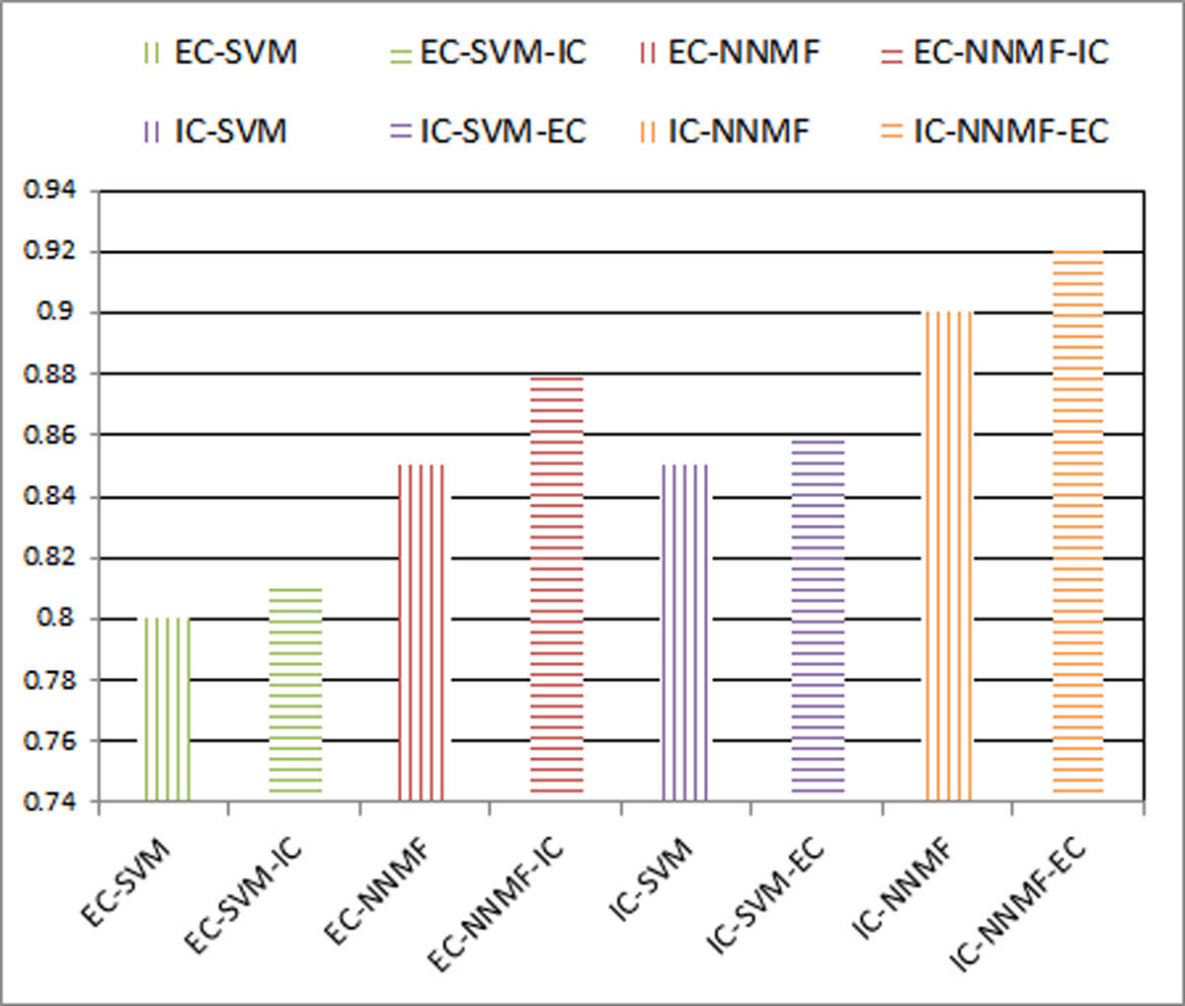
**Impact of enhancement learning**.

As Figure 10 shows that using one set of coded data for the classification of another set of coded data improve the precision. In general, there is about 1-2% increase, if the coded data is used as feature in SVM classification. And there is 2-3% precision increase when the NNMF classification is applied.

## Conclusion

Many previous studies have done general document classification. Injury Text Narrative shows unique characteristics and is different from long document classification. An exclusive study is carried out in this paper with the classifiers particularly suitable for sparse high dimensional datasets.

In the pre-processing step, a soundalike word correction approach is adopted to correct the common errors caused by misunderstanding of correct spelling of words. Some stop-words related to output class such as off/from are combined with the previous word as a phrase. Keeping all terms appearing in the dataset is expensive and unnecessary. An EM clustering approach is adopted to decide the terms cut-off criteria.

In the experiments, various classifiers are compared. NNMF with SVM classifier achieves the best performance. For the traditional classifiers, SVM always achieves higher performances under the tested dataset and the Model-based classifier is superior to memory-based classifier. As Kappa statistic and AUC are less sensitive than general evaluation metric under the imbalanced dataset, they are employed in our experiments. The amount of increasing training size is not at the same pace as the performance increases, thus choosing the correct size of the training set can reduce the resources needed. Top-N terms should not be arbitrarily removed as some frequent words may be the key feature for identifying classes. Guidance from experts is desirable. Binary weighting works better than TF-IDF and TF in short document classification. K should not be the universal number, even though the same input text is used. For different classes, different size of K should be adopted. The inclusion of one coded data helps in improving the classification accuracy of another coded data.

This study used specialised injury surveillance data (which is only available for a small number of emergency departments) to evaluate the feasibility of machine learning and to identify the most accurate methods for classifying narrative text. The findings from this research can be applied to routine administrative emergency department data (which is available for the majority of emergency departments) to classify causes of injuries from presenting problem text to enable broader capture and classification of injury data beyond specialised collections. Developing automated accurate methods for categorising unstructured emergency department text will provide a valuable resource for injury researchers seeking to understand the pattern of causes of injuries treated in emergency departments.

## Competing interests

The authors declare that they have no competing interests.

## Authors' contributions

LC was responsible for designing and undertaking the experiments, analysing the results from said experiments and drafting the manuscript. RN was responsible for reviewing, providing guidance and feedback with relation to the experiments and reviewing draft versions of this manuscript. KV was responsible for designing the overall research project, providing context knowledge, drafting this manuscript's introduction, reviewing drafts of the manuscript, and sourcing the dataset used in the experiments. All authors read and approved the final manuscript.

## Declarations

Publication of this article has been funded by Queensland University of Technology.

This article has been published as part of *BMC Medical Informatics and Decision Making *Volume 15 Supplement 1, 2015: Proceedings of the ACM Eighth International Workshop on Data and Text Mining in Biomedical Informatics. The full contents of the supplement are available online at http://www.biomedcentral.com/bmcmedinformdecismak/supplements/15/S1.
